# A flexible copper MOF as a carboxylate-specific crystalline sponge for structure solution using X-ray and electron diffraction

**DOI:** 10.1039/d5sc05651a

**Published:** 2025-11-10

**Authors:** Russell M. Main, Daniel N. Rainer, Marta Bauzá, Romy Ettlinger, Nicole L. Kelly, Simon J. Coles, Sharon E. Ashbrook, Russell E. Morris

**Affiliations:** a EaStCHEM School of Chemistry Purdie Building, North Haugh St Andrews KY16 9ST UK rmm29@st-andrews.ac.uk; b School of Chemistry and Chemical Engineering, Faculty of Engineering and Physical Sciences, University of Southampton Southampton SO17 1BJ UK; c Department of Chemistry, University of the Balearic Islands Palma de Mallorca E-07122 Spain; d TUM School of Natural Sciences, Technical University of Munich Lichtenbergstr.4 85748 Garching b. München Germany

## Abstract

A new metal–organic framework (MOF) comprising copper and 2,3-dihydroxyterephthalate (2,3-dhtp) has been prepared using solvothermal synthesis. The solid (chemical formula of the as-made material): Cu_12_(dhtp)_4_(H_2_dhtp)_3_(CH_3_CO_2_)_2_ 2DMF·10H_2_O is flexible in that its pore size adapts to match the size of guest molecules that are adsorbed. Carboxylate-containing molecules of different sizes (acetate, benzoic acid and ibuprofen) can be accommodated within the pores of the material and are coordinated to a dimeric copper unit. The localisation of the adsorbate guest molecule, the mode of binding and relatively low symmetry of the MOF allows the system to be used as a crystalline sponge. The crystal structure determination of the as-synthesised acetate-bound MOF was accomplished using single-crystal X-ray diffraction using a synchrotron source, while the benzoate- and ibuprofen-bound structures were solved using electron diffraction. A more practical adsorbent can be formulated by growing the MOF on a cotton fabric substrate, and this is shown to adsorb ibuprofen in a similar manner to the powdered MOF.

## Abstract 2

A new, flexible metal–organic framework has been prepared from copper and 2,3-dihydroxyterephthalate which acts as a crystalline sponge and adsorbent.

## Graphical abstract

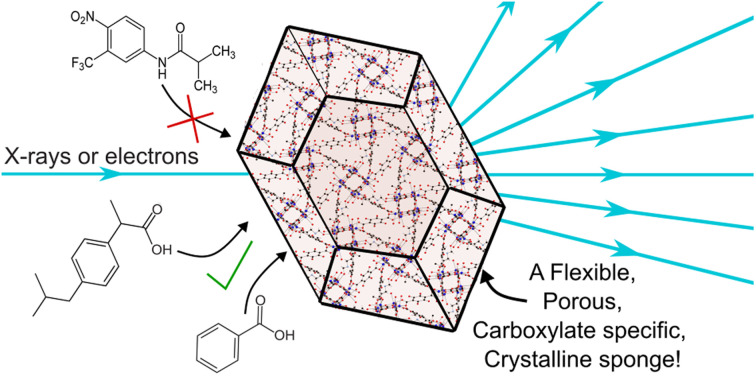

## Introduction

The structure and connectivity of a compound is of paramount interest to all chemists, providing detail on its material properties, reactivity and identity. There are many spectroscopic techniques that can provide information on functional groups and connectivity, but it is single-crystal diffraction that generates the most accurate and complete structural model of a compound and is the gold standard analytical technique for structure determination.^1–3^

The structures of crystalline materials can be determined in several different ways. X-ray diffraction (XRD), first discovered in 1912,^4^ has been the powerhouse of structure elucidation for the last 100 years. Though the most accessible sources of X-rays are those found in in-house machines, synchrotrons can provide high flux and tuneable X-rays perfectly suited for single-crystal X-ray diffraction.^5^ Another method for structure determination is electron diffraction which was first discovered in 1927, confirming de Broglie's theory on electron wave/particle duality.^6^ Though used for structure solution since 1937 it is only relatively recently that structure solution using this approach has become an accessible analytical method.^7,8^ There are now custom built electron microscopes designed for three-dimensional single-crystal electron diffraction (3D ED), which allow for rapid and accurate structure solution on particles too small for traditional X-ray sources.^9^ Neutron diffraction, developed in 1944, can also be used for structure solution, but the requirement for nuclear reactors or spallation sources to produce the neutrons limits its use to large facilities.^10^

However, all these techniques require a crystalline material, and this is not always achievable. For instance, some compounds form naturally as oils, solidify in an amorphous way or there is an insufficient quantity to crystallise the compound.

The crystalline sponge (CS) method pioneered by Fujita and co-workers overcomes this by exploiting the fact that a molecule can be adsorbed into a crystalline host, and the molecule's structure within the host obtained using single-crystal diffraction.^11^ If the guest molecule is sufficiently ordered for unambiguous identification from the diffraction data then the CS method has been successful. To achieve this the host material should have several features. First, and most obvious, it should be crystalline. Second, since extensive disorder of the guest molecule is to be avoided the overall symmetry of the host should be fairly low. This is because high symmetry promotes multiple possible orientations of guest molecules, which makes unambiguous identification of adsorbed species from model refinement against diffraction data much more challenging. Thirdly, to promote guest ordering there is likely a need for strong guest–host interactions. These are fairly stringent criteria and so there are actually very few materials that have proven to be successful CS hosts.^12^ By far the most prevalent is the zinc iodide tris(4-pyridyl)-1,3,5-triazine metal–organic framework (MOF) first used by Fujita's group. This host has been used in successful CS experiments using both X-ray and electron diffraction.^11,13^ The advent of electron diffraction as a tool for structure solution and crystallography is an important development that has enabled the use of much smaller crystals than is possible using X-ray diffraction.^14,15^ For the CS method the use of small crystals has a potential advantage in terms of ease of diffusion of molecules into the host.

Metal–organic frameworks (MOFs) are a varied, exciting and rapidly growing class of materials.^16^ They can be synthesised from a range of metal ions and organic linkers to form crystalline porous frameworks. The chemistry of MOFs can be altered to increase surface areas or change reactivities by using the techniques of reticular chemistry. This allows MOFs to show utility in many fields.^17^ Several of the most well-known MOFs exhibit flexible behaviours,^18^ such as breathing and gate opening, as a response to external stimuli including heat, pressure or solvent molecules.^19^ This leads to a diverse range of potential applications including intermediate sieving^20^ and controlled drug release.^21^

Of the many different applications investigated using MOFs, one area in which they excel is the adsorption of small molecules, from gases^22,23^ to drug molecules.^24^ One interesting and potentially very useful consequence of this adsorption is the potential for MOFs to be used as a crystalline sponge (CS).^25^ MOFs have shown that they are capable of performing this application, including through coordinative alignment and supramolecular docking.^26,27^ However, there are, as yet, surprisingly few examples of MOFs being used in this way.^12,28^

MOFs can also be used in a variety of other applications such as drug delivery, whereby drugs are loaded into a MOF and released by a trigger such as moisture or acidity.^29,30^ However, for use in biomedical applications the toxicity must be considered, with the stability of the MOF and metal choice being important.^31^ MOFs can also be used to capture pollutants from the atmosphere or solution such as volatile organic carbons (VOCs),^32^ polyfluoroalkyl substances (PFAS),^33^ agrochemicals,^34^ viruses^35^ and medicines.^36^ Moreover, the application of MOFs can be broadened by the fabrication of composites.^37^ MOFs can be engineered through their incorporation into different substrates, combining the advantages of both parts and allowing for their better practical use. Different natural cellulose-based materials have been embedded with MOFs to create MOF@cellulose hybrids.^38^ In particular, the use of cotton as a substrate has resulted in promising candidates for different applications.^39,40^

We present here a new flexible MOF based on copper and 2,3-dihydroxyterephthalic acid (2,3-dhtp). The structures of both the open and narrow pore form have been solved with single-crystal X-ray diffraction (scXRD) and the properties of the MOF characterised. In addition, carboxylate-containing molecules of different sizes have been adsorbed into its structure and electron diffraction used to determine their location and structure. This MOF shows a particularly favourable binding site for carboxylate-containing molecules and its low symmetry makes it a suitable candidate as a crystalline sponge. Furthermore, the MOF was synthesized *in situ* on a cotton fabric, resulting in a flexible and uniformly coated composite. The potential loading capacity of the prepared hybrid was also proved, with Ibuprofen as a proof of concept molecule, opening up the possibility of using this composite as an engineered adsorbent.

## Results

### Synthesis and structure of SIMOF-5

**Fig. 1:**
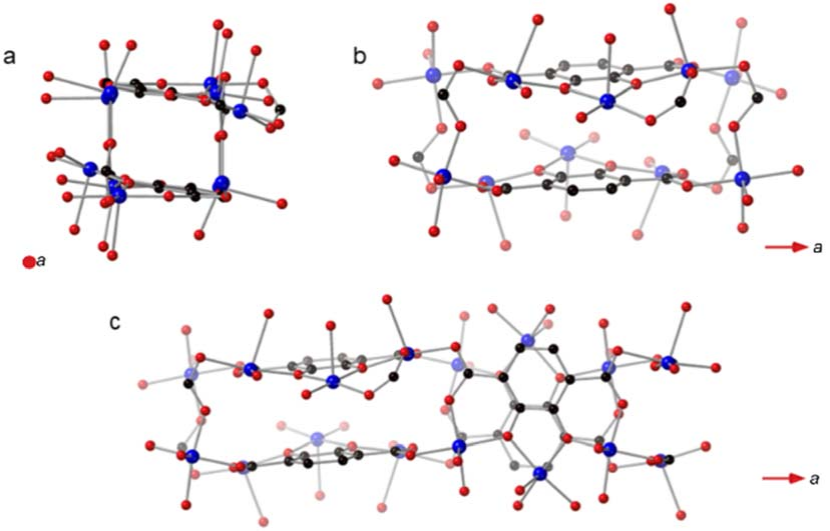
(a) The cuboidal SBUs in SIMOF-5 are arranged to form chains that run parallel to crystallographic *x* axis. (b) One half of the SBU comprises six copper atoms, three of which are linked by one fully deprotonated 2,3-dhtp molecule to form one face of the cuboid while the opposite face is related by crystallographic symmetry. (c) The full SBU is formed from another half SBU rotated approximately 90° with respect to the first half. The two half SBUs have the same connectivity but are not related by crystallographic symmetry. Key: blue = copper, red = oxygen, black = carbon. Note hydrogen atoms are not shown.

**Fig. 2:**
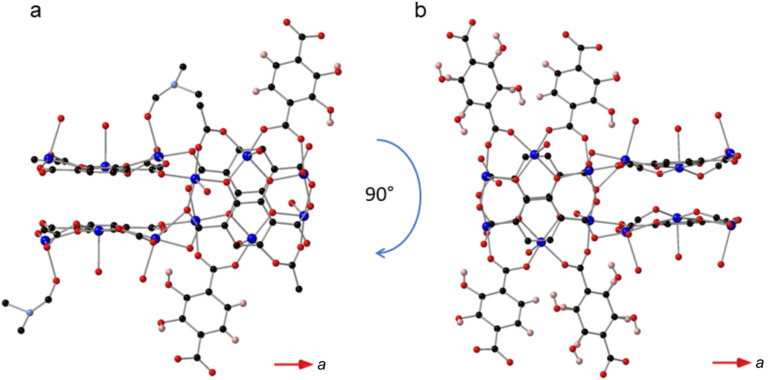
Two views of one SBU from SIMOF-5 showing how (a) one half is connected through one pair of symmetry-related 2,3-dhtp linkers with the other sites taken up by acetate groups; also shown is a pair of symmetry-related coordinated DMF molecules. (b) The same SBU rotated by ∼90° shows the other half of the SBU is connected through two pairs of 2,3-dhtp linkers. Note that one pair of 2,3-dhtp linkers in (b) is 50 : 50 disordered while the other pair is ordered. Key: blue = copper, red = oxygen, black = carbon, pink = hydrogen.

**Fig. 3:**
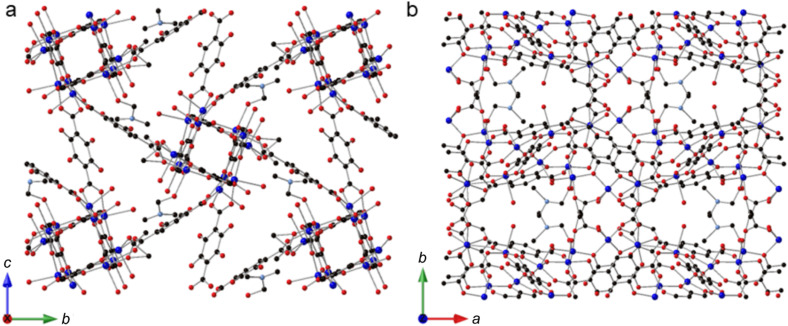
(a) A view of SIMOF-5 parallel to the crystallographic *x* axis showing how the chains of cuboidal SBUs are connected to form small triangular pores. (b) A view of SIMOF-5 parallel to the *z* axis showing the tear drop shaped pores containing the coordinated DMF and water.

To produce new MOFs with interesting structural features the choice of organic linker and metal is crucial. In this work the linker 2,3-dhtp was chosen, because of its lack of an inversion centre,^41,42^ and combined with copper(ii), as this is a highly labile metal ion, so chosen as a candidate to increase the chances of generating a flexible framework.^28,43^ Reacting copper acetate and 2,3-dhtp at 60 °C in DMF yielded plate-like nanocrystals of a new MOF denoted as SIMOF-5 (St Andrews MOF). Synthesising the material in a 1 : 1 solvent ratio of DMF : water yielded the formation of single crystals (Fig. S1). Their structure was elucidated using the synchrotron source at Diamond Light Source (Fig. 1–3) using scXRD.

SIMOF-5 crystallises in the *P*2_1_/*c* space group with unit cell parameters: *a* = 17.071(3) Å, *b* = 20.985(4) Å, *c* = 16.560(3) Å, *β* = 115.245(3)° and a unit cell volume of 5366(1) Å^3^. Its formula is Cu_12_(dhtp)_4_(H_2_dhtp)_3_(CH_3_CO_2_)_2_.2DMF·10H_2_O where dhtp is the fully deprotonated 2,3-dhtp C_6_H_2_O_2_(CO_2_)_2_^4−^, H_2_dhtp is only deprotonated at the carboxylate groups C_6_H_2_(OH)_2_(CO_2_)_2_^2−^, CH_3_CO_2_ is an acetate anion and DMF is the neutral solvate (CH_3_)_2_NCHO.

SIMOF-5 exhibits an extremely complex crystal structure built from very unusual secondary building units (SBUs) with a cuboid shape that form chains that run parallel to the crystallographic *a* axis (Fig. 1a). One half of the SBU contains six copper atoms split into three symmetry-related pairs (Fig. 1b) that form opposite faces of a cuboid. The copper atoms are each five coordinated in a pseudo square pyramidal geometry. The axial position in the square pyramid is taken by oxygen atoms from solvent molecules and all point outwards from the cuboid while the equatorial oxygen atoms come from the 2,3-dhtp or acetate molecules. The Cu–O interatomic distances range from 1.86(1) to 2.51(9) Å. Within the SBU the 2,3-dhtp molecules are fully deprotonated and fully connected to copper atoms to form the remainder of the faces of the cuboid. The other half of the SBU is very similar in terms of connectivity but is rotated approximately 90° with respect to the first to form the overall cuboidal SBU shown in Fig. 1c. The SBUs then repeat parallel to the *a* axis to form the chains shown in Fig. 1a.

At first sight the two halves of the SBU look like they could be related by crystallographic symmetry because they are so similar in internal connectivity. However, they differ significantly in how they are connected in the three-dimensional structure. Fig. 2 shows how one half of the SBU is connected to four 2,3-dhtp molecules that in turn connect to other SBUs in neighbouring chains. In contrast, the other half of the SBU, rotated approximately 90° to the first half, is only connected to other chains of SBUs through two 2,3-dhtp linkers, with the remaining two carboxylate sites taken up by acetate groups. In contrast to the 2,3-dhtp ligands that lie within the SBU, the 2,3-dhtp units that link different chains of SBUs only coordinate through the carboxylate groups, with the catechol hydroxides remaining protonated. It is also interesting to note that one symmetry-related pair of 2,3-dhtp linkers is disordered across the C_2_ symmetry axis of the 2,3-dhtp molecule while the other two pairs are ordered. Finally, one molecule of DMF per SBU could be located from the diffraction data, coordinated to a Cu atom (Cu_4_).

The difference in connectivity between different parts of the SBUs leads to a very interesting but complex overall three-dimensional MOF structure. Fig. 3a shows a view of the structure parallel to the crystallographic *a* axis showing how the chains of the cuboidal SBUs, which also run parallel to the *a* axis, are connected by 2,3-dhtp linkers to form small triangular channels. Fig. 3b shows a view parallel to the crystallographic *c* axis. This view shows pores that are narrower at one end compared to the other (termed here a “tear drop” shape). These pores contain the coordinated solvent molecules (DMF). There are no pore features visible when viewed parallel to the *b* axis. The complex packing of this framework can be seen in Fig. S2 which shows a simplified nodal representation of the framework with the two pore environments highlighted.

### The structure of desolvated SIMOF-5

**Fig. 4:**
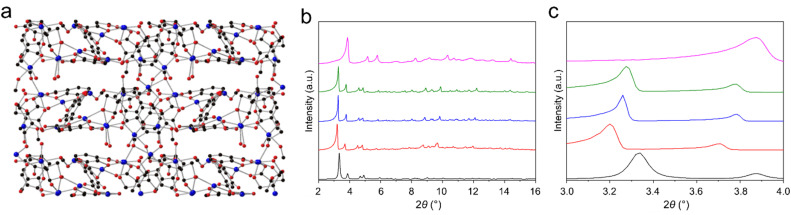
(a) A view of the desolvated SIMOF-5 structure in the same direction as Fig. 3b showing how the flexibility in the overall structure leads to a reduction in the size of the teardrop shaped pores. (b) Powder X-ray diffraction patterns (Mo Kα radiation, room temperature) showing the predicted patterns (from bottom to top) based on the single crystal structure of the as made material (black), the as-made sample after stirring in DMF (red), after drying at room temperature (blue), after drying at 60 °C for 10 minutes (green) and after solvent exchange and drying at 60 °C to form the desolvated material (pink). (c) Low angle regions of the patterns shown in (b) indicating how the drying conditions affect the position of the reflections.

Removing the guest molecules from inside the pores of SIMOF-5 involved solvent exchange with ethanol followed by thermal activation at 60 °C. Powder XRD showed a significant shift in the positions of the reflections in the pattern (Fig. 4b), indicating a reduction in the size of the unit cell. Unfortunately, this process had a detrimental effect on the quality of the single crystals that precluded the possibility of collecting high-quality structural data. However, scXRD using synchrotron radiation did allow data of sufficient quality for the determination of basic structural information to be collected. The desolvated MOF crystallises in the *P*2_1_ space group with unit cell parameters *a* = 15.659(4) Å, *b* = 15.511(5) Å, *c* = 17.028(3) Å, *β* = 116.83(2)° and a volume of 3690(2) Å^3^ (Fig. 4a). Its formula is Cu_12_(dhtp)_4_(H_2_dhtp)_3_(CH_3_CO_2_)_2_·4H_2_O but it must be noted that due to the data quality there maybe some error in the solvent content. The resultant crystal structure shares many characteristics with the solvated phase. It consists of the same basic SBU connected in the same manner as in the solvated material. The bridging linkers bind these chains together forming triangular pores down the crystallographic *c*-axis that are smaller than those observed in the solvated structure (Fig. S2). The tear drop pores are now absent with only a small space between the copper rich layers. There are both 4- and 5-coordinate Cu atoms with some residual solvent binding and located in the small pores – this indicates that the crystals are not quite fully desolvated. Successful refinement required significant use of constraints and restraints to model the linkers sensibly. However, the main features of the structure are still clearly visible. The peak positions from the experimental powder pattern, though broad, match those predicted from this structure supporting the veracity of this model (Fig. S3). The reversible flexibility of the framework can be shown by exposing the desolvated MOF to polar solvents such as DMF, and this leads to the reformation of the original SIMOF-5 structure (Fig. S4). The flexibility of the material is evident in the powder XRD patterns where the level of solvent present has a marked effect on the positions of the low-angle reflections, and different drying conditions produce materials with different unit cell sizes (Table S1). The largest change to the material occurs only after solvent exchange with ethanol and drying at 60 °C, which produces the desolvated phase (Fig. 4b and c). Thermal gravimetric analysis (Fig. S5) and N_2_ adsorption profiles (Fig. S6) for SIMOF-5 confirm the molecular formula and surface area suggested by the single-crystal structures.

### SIMOF-5 as a carboxylate selective crystalline sponge

Fujita's crystalline sponge method is recognised as a novel method for structural characterisation of molecules that are contained within the pores of crystalline porous solids.^12,44^

**Fig. 5:**
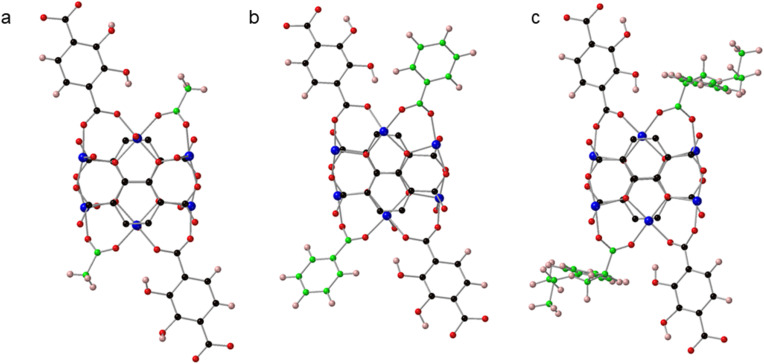
(a) Portion of the SIMOF-5 structure derived from single crystal X-ray diffraction showing the binding of the acetate groups (b) the same portion of the SIMOF-5 structure derived from electron diffraction after loading with benzoic acid and (c) with isobutylphenylpropionate (ibuprofen). Key: blue = copper, red = oxygen, black = carbon, pink = hydrogen. Note the carbon atoms of the acetate (a), benzoate (b) and isobutylphenylpropionate are shown in green. Hydrogens atoms on the 2,3-dhtp within the SBUs are not shown for clarity.

The structure of SIMOF-5 described above has several features that make it of potential interest as a CS host MOF. It is monoclinic (and so low symmetry) and crystalline, and it can be made in crystal sizes suitable for both electron and X-ray diffraction. Perhaps of most importance is that the structure shows a potential site that is geometrically ideal to bind carboxylate-containing species – in the as-made material this is taken up by acetate. Just as important is the fact that these carboxylate-binding sites are relatively dilute in the structure, which lowers the possibility of any guests being disordered by having multiple possible orientations. Given the potential for use as a CS host, carboxylate-containing molecules of different sizes, *i.e.*, acetate, benzoic acid and isobutylphenylpropionic acid (more commonly known as ibuprofen) were identified as proof of concept guests. Ethanol-exchanged MOF material was placed in an ethanolic solution of the guest molecule and left for 3 days. The crystals were then collected by filtration and electron diffraction data collected at the National Electron Diffraction Facility at the University of Southampton, UK. The structure of the solid treated with benzoic acid revealed that carboxylate exchange occurred successfully and electron diffraction studies revealed that the benzoate molecule binds at the same site as the acetate groups (Fig. 5). Interestingly, the diffraction experiment also revealed that there were ordered, uncoordinated benzoic acid molecules in the pore space of the MOF (Fig. S7). This ordering within the MOF causes a symmetry change and an increase in the unit cell size of the overall structure (Table S2), which can also be identified in the PXRD patterns where the low-angle reflections are split (Fig. S8). In contrast, the material loaded with isobutylphenylpropionic acid retains the symmetry of the as-made sample but shows only a slight increase in the unit cell size, as would be expected given the size of the guest molecule. As for the acetate and benzoate cases, diffraction reveals that isobutylphenylpropionate binds to same two copper atoms in the SBU and the model can be refined successfully to unambiguously identify this molecule also. There are no ordered isobutylphenylpropionic acid guest molecules that are not coordinated. Due to the vacuum conditions required for electron diffraction the solvent found in these systems (modelled as water) has a range of occupancies (0.33–1.00) in different sites that will be different under atmospheric conditions. However, the formulae of the two guest loaded structures can be well approximated as Cu_12_(dhtp)_4_(H_2_dhtp)_3_(benzoate)_2_(benzoic acid)_1.8_·*X*H_2_O and Cu_12_(dhtp)_4_(H_2_dhtp)_3_(isobutylphenylpropionate)_2_·*x*H_2_O.

The fact that the three carboxylate-containing guest molecules are bound at the same site in the loaded materials and are ordered, despite being of very different size, flexibility and symmetry, indicates that SIMOF-5 is likely a general CS host for molecules that can bind to the specific sites in this way. This coordinative alignment strategy has been seen before in CS hosts such as MOF-520.^26^ As a control to test this further, the molecule 4′-nitro-3′-trifluoromethyl-isobutyranilide (commonly known as the drug flutamide) was adsorbed into the MOF. This molecule has no carboxylate group and does not bind to the copper sites, and despite there being evidence of bulk flutamide adsorption from IR spectroscopy, TGA and other techniques that show the flutamide molecules were in the pores of the MOF (Fig. S8–14), no ordered molecules could be located using electron diffraction. Evidence for the presence of the guest molecule in the particular crystal used for structure determination was obtained by EDS, which indicated the presence of fluorine (see Fig. S15). This demonstrates the carboxylate-selectivity of SIMOF-5 further. Other characterisation methods are all consistent with the structures as shown. For example, infrared spectroscopy, PXRD, thermogravimetric analysis and electron paramagnetic resonance (EPR) spectroscopy can demonstrate the presence of the guest molecules (Fig. S8–12). The importance of the copper coordination chemistry is confirmed by the EPR experiments which show a clear difference between the spectrum of the acetate-containing solid and one exposed to isobutylphenylpropionic acid. However, exposure to 4′-nitro-3′-trifluoromethyl-isobutyranilide causes no change in the spectra (Fig. S10). Furthermore, the presence of the guest molecules can be observed from their release in ethanol (Fig. S13, 14 and Table S3). Full details of these experiments can be found in the SI.

### SIMOF-5/cotton composites for adsorption

A major component of modern MOF research is the development of how to formulate MOFs into materials that can be used more easily in applications. While powdered crystalline MOFs may be of use in certain situations (such as the crystalline sponge applications described above) many other applications might require different formulations of MOFs, for example as composites with fabrics or polymers. Adsorption applications to remove potential harmful pollutants are particularly of interest for cotton/MOF composite fabrics,^45^ and there are now several examples for the removal of drug molecules from the environment.^39,46^

**Fig. 6:**
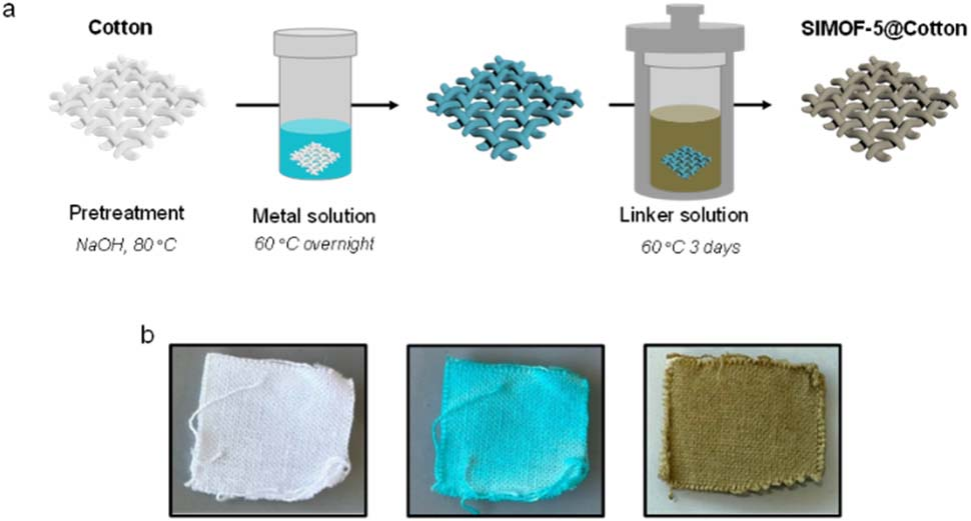
(a) Schematic preparation of SIMOF-5@cotton composite. (b) Photographs of pristine cotton fabric, copper acetate coated cotton fabric and SIMOF-5@cotton composite.

Inspired by our previous work, SIMOF-5 can be incorporated into a composite material by growing it upon a cotton matrix.^35^ The preparation process of SIMOF-5@cotton composites is shown schematically in Fig. 6. First, cotton fibres were pretreated with NaOH to activate the surface of the fabric. Cotton is composed of cellulose, a natural polysaccharide and treatment with hydroxide can induce partial negative charges on the surface of cellulose, making it more reactive.^47^ After the preconditioning of the cotton fabric, the substrates were immersed in a copper acetate solution, which allowed for the incorporation of copper through the fibres of cellulose, as indicated by the characteristic blue colour of this metal on the fabric. Then, the linker solution was added to the mixture to achieve a uniform growth of brown SIMOF-5 along the fibre. The amount of SIMOF-5 supported on 73 ± 2 mg of cotton was 13.7 ± 0.5 mg, so therefore the composite is approximately 16 wt% MOF. Images of the fabric before and after the growth are presented in Fig. 6b.

**Fig. 7:**
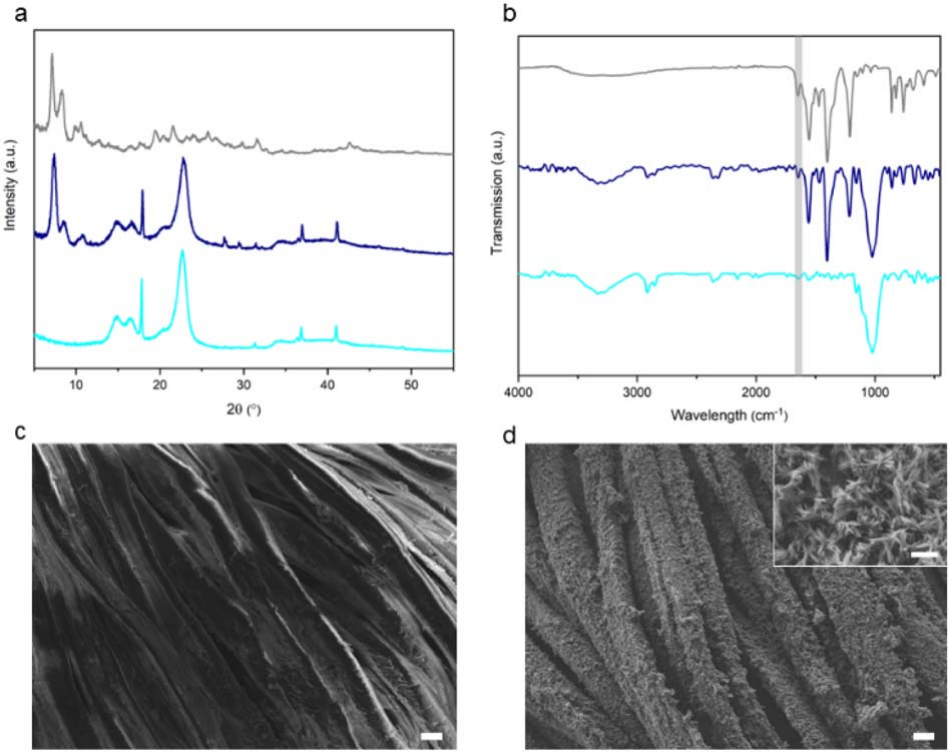
(a) XRD patterns, obtained with Cu Kα radiation, and (b) FTIR spectra of cotton (light blue), SIMOF-5@cotton composite (dark blue) and SIMOF-5 swollen (grey). Peaks at 27.6, 29.3 and 32.0° correspond to tape, while the peak at 17.8° is from PTFE (used in sample holder). DMF band is highlighted in grey. SEM images of (c) pristine cotton and (d) SIMOF-5@cotton. Main scale bars are 10 µm, inset scale bar is 1 µm.

The XRD pattern of the SIMOF-5@cotton confirmed the presence of SIMOF-5 material on the fiber (Fig. 7a). New peaks were clearly detected on the composite, matching well with the simulated pattern of the swollen SIMOF-5, along with peaks at 14.7, 16.5 and 22.6° from the pristine cotton fibers, demonstrating that SIMOF-5 was successfully grown on the substrate. To further confirm the loading of SIMOF-5 using this procedure, FTIR was also carried out (Fig. 7). For cotton, the broad band at 3330 cm^−1^ is attributed to the hydroxyl groups (OH^−^) of cellulose, whilst an intense band at 1029 cm^−1^ is related to the C–O stretching vibration of that compound.^48^ The FTIR spectrum of SIMOF-5@cotton shows bands from both the cotton substrate and SIMOF-5, indicating successful formation of the composite (Fig. 7b). The SEM of bare cotton and SIMOF-5@cotton composite are presented in Fig. 7c and d. The images reveal the cotton fibres have been uniformly coated by SIMOF-5, forming a dense layer of needle-shaped crystals. The mechanical stability of the composite was also tested and the SIMOF-5@cotton composite remained mainly unmodified even after 30 cycles of adhesive tape and sandpaper abrasion tests (Fig. S16), demonstrating good robustness and mechanical resistance of the composite when in contact with an adhesive or rough surface.

We also aimed to explore the potential of the composite as a shaped adsorbent. Given the success of the crystalline sponge work reported above and the fact that it is a common pollutant, isobutylphenylpropionic acid (ibuprofen) was selected as a model compound for loading onto SIMOF-5@cotton. The composite was exposed to a concentrated ethanolic solution of isobutylphenylpropionic acid and after soaking for several days, the composite was recovered, dried and characterised.

The diffraction pattern (Fig. S17a) of Ibu@SIMOF-5@cotton presents peaks at 6, 16 and 20° from ibuprofen, along with those characteristic of the loaded MOF and bare cotton, indicating its adsorption in both the MOF and the cotton. Moreover, the observed intense band at 1710 cm^−1^ in the FTIR spectrum (Fig. S17b) of the composite confirms the presence of this molecule and its successful loading on the modified cotton fibres. TGA (Fig. S18) shows that plain cotton can adsorb ibuprofen but SIMOF-5@cotton nearly doubles the weight percentage of loaded ibuprofen confirming the beneficial effect of adding the MOF.

## Conclusion

SIMOF-5 was successfully synthesised from 2,3-dhtp and copper(ii) ions. Its relatively low symmetry, guest responsive flexibility and low density of selective carboxylate binding sites make it an ideal crystalline sponge for carboxylate-containing molecules. This was successfully demonstrated using acetic acid, benzoic acid and isobutylphenylpropionic acid. The structure of SIMOF-5 adapted to accommodate the differently sized molecules allowing for the structure of each to be successfully determined. Molecules without a carboxylate binding motif, such as 4′-nitro-3′-trifluoromethyl-isobutyranilide, did not bind to the selective site and their structure cannot be determined through diffraction techniques. The crystalline sponge method first developed by Fujita^11^ and co-workers is a powerful technique for the structural elucidation of compounds that cannot be crystallized or are unstable under collection conditions and therefore has potentially wide ranging applicability. This MOF not only presents a new addition to the family of crystalline sponge materials, using both electron and X-ray diffraction, but also presents the possibility to selectively bind a desired molecule from a mixture of compounds. A selective crystalline sponge would be highly desirable allowing for structural determination of impure product streams, reducing the time and energy needed for extraction.

Furthermore, the synthesis of a SIMOF-5@cotton composite demonstrates how the adsorption capacity of SIMOF-5 may be utilised in, for instance, the adsorption of contaminants from solution. By forming a stable MOF composite that can be handled and shaped safely it expands the uses of the MOF beyond that of the parent powder.

## Materials and methods

### Synthesis of 2,3-dhtp

Oven dried catechol (7.5 g, 68.1 mmol) and potassium carbonate (20.46 g, 204 mmol) was placed in an autoclave. The autoclave was flushed with a vacuum, N_2_ cycle three times and then charged with CO_2_ to a pressure of 10 bar. The vessel was heated to 230 °C at 50 °C increments and left overnight. The product was cooled, the solid crushed and then suspended in water (300 mL). The liquid was separated using centrifugation at 6000 rpm, and HCl (25 mL) was added. The resultant precipitate was filtered, washed with water and ethanol and dried in on oven overnight to produce a pink powder of 2,3-dhtp.^49^

When required, the 2,3-dhtp was recrystallized from a 1 : 1 water ethanol solvent mix, to produce pale pink crystals.

### Synthesis of SIMOF-5 single crystals

1 mmol of Cu(ii) acetate monohydrate was dissolved in 8 mL water and 300 µL of acetic acid. 1 mmol of 2,3-dhtp was dissolved in 8 mL DMF. The two solutions were mixed in a pressure sealed autoclave and heated to 60 °C for 3 days. The resultant solid was separated *via* filtration and washed with DMF for the swollen form, or DMF and EtOH for the desolvated form. This produced brown crystals of SIMOF-5.

### Synthesis of SIMOF-5 nanoplates

1 mmol of Cu(ii) acetate monohydrate was dissolved in 8 mL DMF and 300 µL of acetic acid. 1 mmol of 2,3-dhtp was dissolved in 8 mL DMF. The two solutions were mixed in a pressure sealed autoclave and heated to 60 °C for 3 days. The resultant solid was separated using centrifugation and washed with DMF for the swollen form, or DMF and EtOH for the desolvated form. This produced brown nanoplates of SIMOF-5.

### Synthesis of SIMOF-5@cotton composites

The synthesis of SIMOF-5@cotton was similar to the one of pristine SIMOF-5 (nanoplates). In this regard, first, a piece of cotton fabric (approx. 2 × 2 cm) was pretreated by soaking in 3M NaOH for 20 minutes at 80 °C, washed with water and dried at the same temperature. The preconditioned cotton was then submerged in a solution containing 1 mmol of Cu(ii) acetate monohydrate, 8 mL DMF and 300 µL acetic acid and heated at 60 °C overnight in a Teflon-lined vessel. 1 mmol of 2,3-dhtp in 8 mL DMF was then added to the previous metallic solution, which also contained the cotton. The final mixture was placed in the oven at 60 °C for 3 days in an autoclave. After cooling down, the SIMOF-5@cotton composite was washed three times with DMF and dried at 80 °C.

### Adsorption of carboxylate-containing molecules

1.5 mmol of adsorbate (ibuprofen or benzoic acid) was dissolved in 6 mL EtOH. 70 mg of MOF was submerged into this solution and the resultant mixture agitated. It was then allowed to stand at room temperature with no stirring for three days.

### Flutamide loading control experiment

For flutamide loading the same procedure was followed with the vial covered in tin foil to reduce light degradation. Then the solvent was removed with a pipette and the solid was dried in a drying oven at 60 °C.

### Adsorption of ibuprofen onto SIMOF-5@cotton

To explore the potential of adsorption of carboxylates onto SIMOF-5@cotton composites a piece of composite was submerged in a solution of 1.5 mmol of ibuprofen dissolved in 6 mL EtOH at room temperature for three days. The composite was taken out and dried at 60 °C.

### Release measurements

3–5 mg of loaded MOF was placed in 15 mL of EtOH. The solutions were kept at room temperature and not stirred. At select time intervals 3 mL aliquots of the solution were taken and their UV/vis spectra obtained on a CARY 60 UV/vis spectrometer from Agilent Technologies. The aliquots were then carefully returned to the parent liquor to avoid dispersing the MOF particles.

### X-ray crystallography

For compound SIMOF-5 as-made, fomblin oil was used to coat a selection of crystals which were then mounted on MiTeGen kapton loops and frozen in liquid nitrogen. The loops were stored in a MiTeGen Unipuck and transported to Diamond Light Source. Data were collected remotely at beam line I19 of Diamond Light Source using double crystal monochromated synchrotron radiation (*λ* = 0.6889 Å) and a Dectris Pilatus 2M pixel-array photon-counting detector.^50^ The data was processed using Apex3.^51^ Subsequently, Olex2 GUI^52^ (with shelXT^53^ as solution and shelXL^54^ as refinement tool) was used for structure solution and refinement, respectively. Obtained crystal structures were visualised using the CrystalMaker software kit.^55^ Non-hydrogen atoms were refined anisotropically and H atoms were refined using a riding model. The metal bound DMF was subject to a SIMU restraint across all atoms of strength 0.02 and distance 2.7 Å. The disordered 2,3-dhtp molecule was modelled with each phenol having an occupancy of 50%, the equivalent hydrogen could not be refined sensibly. The outer carbon of the acetate molecule was disordered with hydrogens only modelled on the major component.

Selected crystals of desolvated SIMOF-5 were mounted on MiTeGen kapton loops with a two part epoxy resin and analysed at 300 K on the three-circle diffractometer equipped with a Pilatus 2M detector in I19-1 beamline, Diamond Light Source. A wavelength of 0.6889 Å was utilized. Data collection were setup using the generic data acquisition (GDA) software and were processed using xia2 (ref. 56) with DIALS^57^ routines. Subsequently, Olex2 GUI^52^ (with shelXT^53^ as solution and shelXL^54^ as refinement tool) was used for structure solution and refinement, respectively. Obtained crystal structures were visualised using the CrystalMaker software kit.^55^ The low quality diffraction (low *I*/*σ* and high *R*_int_) meant constraints were needed on all the organic components. AFIX 66 constraints for the aromatic rings were used as well as FLAT and DFIX restraints for the functional groups. Isotropic displacement parameters were constrained to 0.08 except for some O atoms that could be refined anisotropically. Cu atoms were unconstrained and refined anisotropically. H atoms on SBU based linkers were refined using a riding model. The high level of disorder in bridging organic linkers meant H atoms were not refined.

Powder patterns of SIMOF-5 were obtained with Mo Kα radiation. Initial cell refinement and error calculation was performed with WinXPOW (3.7.0.0)^58^ additional refinement was performed with GSAS II (5084).^59^

### Electron diffraction

The grids were prepared dry, by gently grinding the solid powder between glass slides and depositing it on a holey carbon coated gold grid (200 mesh, Agar Scientific, UK). The grids were then mounted using cryo-transfer on a Gatan Elsa cryogenic holder at a temperature of 175(5) K. Data was also collected at 175(5) K. All data collections were performed on a Rigaku XtaLAB SynergyED operated at 200 kV and equipped with a Rigaku HyPix-ED hybrid pixel array area detector and a JEOL JED-2300 EDS detector. 3D ED measurements were performed in continuous rotation mode using a selected area aperture with apparent diameter of approximately 2 µm in the image plane under optimised beam conditions. Data were collected using CrysAlisPRO (version 1.171.43.118a for Ibu@SIMOF-5, 1.171.44.67a for Flt@SIMOF-5 and 1.171.44.70a for BA@SIMOF-5).^60^

In all cases, data were collected from 9–14 particles, some of which were indexable in the respective unit cells (see Tables S4–S6), however, for all three samples only a single collection was of sufficient quality for structure determination.

The datasets were in each case individually indexed, integrated, and scaled using CrysAlisPRO (version CrysAlisPro 1.171.44.81a for Ibu@SIMOF-5 and 1.171.44.70a for Flt@SIMOF-5 and BA@SIMOF-5)^60^ and SCALE3 ABSPACK implemented therein. All three structures were solved using ShelXT^61^ and refined in the kinematic approximation using Olex2.refine^62^ as implemented in Olex2, version 1.5-ac7-013 (compiled 2025.01.02 svn.rf662f148 for Rigaku Oxford Diffraction, GUI svn.r7109)^52^ using published scattering factors.^63^ An extinction correction was applied in each case to broadly account for the impact of multiple scattering with further omission of particularly outlying reflections in the final stages of the refinement where necessary.

In all cases, several restraints on bonds and ADPs had to be applied to arrive at a physically sensible model. Hydrogens were placed at geometrically constrained positions at tabulated distances from neutron diffraction data and refined using riding isotropic displacement parameters.

Complete experimental and refinement data are contained in the deposited CIFs along with structure factors and an embedded .res file, deposited in the CSD with CCDC reference codes CCDC 2415257–2415259. Tables S4–S6 report experimental parameters from the associated datasets.

### Mechanical testing

The mechanical resistance and durability of the SIMOF-5@cotton composite was evaluated through adhesive tape peeling and sandpaper abrasion tests. For the adhesive test, a strip of tape was adhered to the surface of the composite and repeatedly peeled off. The sandpaper test was conducted using 1200-mesh sandpaper and various abrasion cycles were performed placing 200 g weight above the composite. Each abrasion cycle involved 10 cm of friction.

### Further analysis

PXRD patterns were recorded on a STOE STADI/P diffractometer using Mo Kα1 radiation at room temperature in capillary Debye–Scherrer mode. The cotton samples were measured using a PANalytical Empyrean diffractometer using Cu Kα1 radiation at room temperature in reflection, Bragg Brentano,Theta-2Theta mode. Calculated patterns were generated using the Mercury software package.^64^ FTIR spectra were obtained using a Shimadzu IRAffinity-1S spectrometer (4000–400 cm^−1^). TGA was performed using a STA780 with a crucible and a temperature ramp of 10 °C min^−1^ under air flow of 30 mL min^−1^. N_2_ adsorption isotherms were recorded on a Micromeritics Tristar ii Surface Area and Porosity Instrument. Samples were added to a frit tube and activated *in vacuo* (∼3 × 10^−5^ mbar, 16 h) prior to the measurement. SEM micrographs were collected using a JEOL IT800 at a working distance of 4 mm and low operating voltages (2–5 kV) to ensure sensitive mapping of the surface. The powder samples were placed on aluminium tape. EPR measurements were performed on a continuous wave Bruker EMX plus spectrometer at X-band (9.5 GHz) at room temperature. Experiments were undertaken using 1 mW microwave power, 0.3 mT modulation amplitude, 100 kHz modulation frequency, 800 mT field sweep centred at 450 mT with 26 667 points resolution, a time constant of 10.24 ms and conversion time of 9.90 ms. Samples were packed in a 4 mm Suprasil EPR tube.

## Author contributions

RMM: conceptualization, investigation, formal analysis, writing – original draft; DNR: investigation, formal analysis, writing – original draft; MB: investigation, writing – original draft; RE: investigation, writing – review & editing; NLK: investigation, writing – review & editing; SJC: writing – review & editing, supervision, funding acquisition; SEA: writing – review & editing, supervision, funding acquisition; REM: conceptualization, writing – original draft, supervision, resources, funding acquisition.

## Conflicts of interest

There are no conflicts to declare.

## Supplementary Material

SC-OLF-D5SC05651A-s001

SC-OLF-D5SC05651A-s002

## Data Availability

The research data underpinning this publication can be accessed at https://doi.org/10.17630/3a837046-1fe7-43a2-9179-e8f1e87d819a. 3DED raw data are deposited at https://doi.org/10.5281/zenodo.14617589. CCDC 2404308, 2404310 and 2415257–2415259 contain the supplementary crystallographic data for this paper.^65*a*–*e*^ Supplementary information: further characterisation and crystallographic information for this work. See DOI: https://doi.org/10.1039/d5sc05651a.
