# Supplementary material for: A flexible copper MOF as a carboxylate-specific crystalline sponge for structure solution using X-ray and electron diffraction
Source: Chem Sci. 2025 Nov 10;17(2):1116–26. doi: 10.1039/d5sc05651a (PMC12628275; doi:10.1039/d5sc05651a)
Supplement: SC-017-D5SC05651A-s001 [file SC-017-D5SC05651A-s001.pdf]

Supplementary Information (SI) for Chemical Science.
This journal is © The Royal Society of Chemistry 2025

A flexible copper MOF as a carboxylate-specific crystalline sponge for structure solution using X-ray and electron diffraction.

Supplementary Information

Russell M. Main[1], Daniel N. Rainer[2], Marta Bauzá[1,3], Romy Ettlinger[1,4], Nicole L. Kelly[1], Simon J. Coles[2], Sharon. E. Ashbrook[1] and Russell E. Morris[1]

1. EaStCHEM School of Chemistry, Purdie Building, North Haugh, St Andrews KY16 9ST, UK;
2. School of Chemistry and Chemical Engineering, Faculty of Engineering and Physical Sciences, University of Southampton, Southampton, SO17 1BJ, UK
3. Department of Chemistry, University of the Balearic Islands, Palma de Mallorca E-07122, Spain
4. TUM School of Natural Sciences, Technical University of Munich, Lichtenbergstr.4; 85748 Garching b. München, Germany

Contents

Supplementary characterisation of SIMOF-5. 2
Supplementary information on SIMOF-5 loaded with different molecules. 6
Supplementary data on SIMOF-5@cotton........ 11
Further refinement details 13

Supplementary characterisation of SIMOF-5.

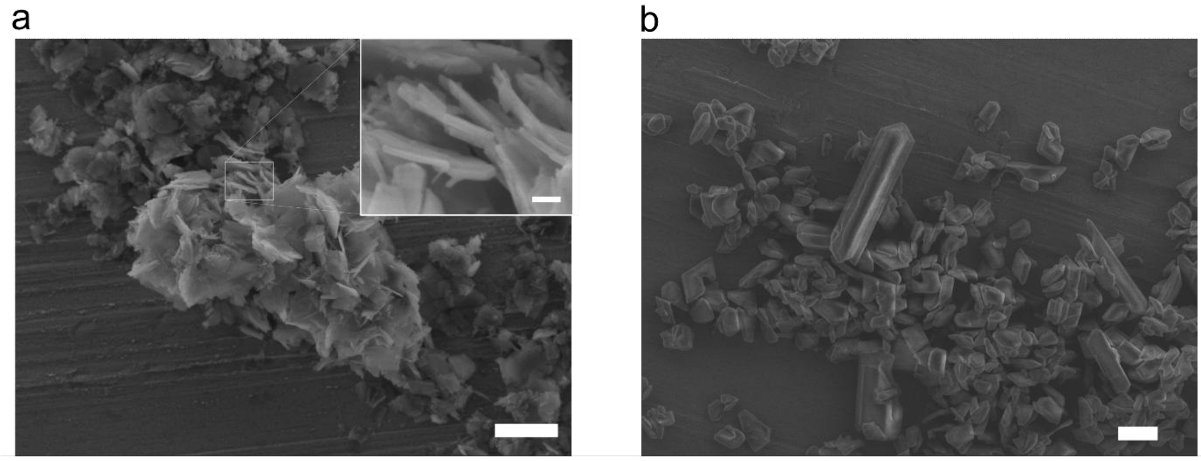

Figure S1: a) SEM image of SIMOF-5 nanoplates synthesised in DMF. b) SEM image of SIMOF-5 single crystals synthesised in 1:1 DMF:water. Main scale bars are 2 μm, inset scale bar is 200 nm.

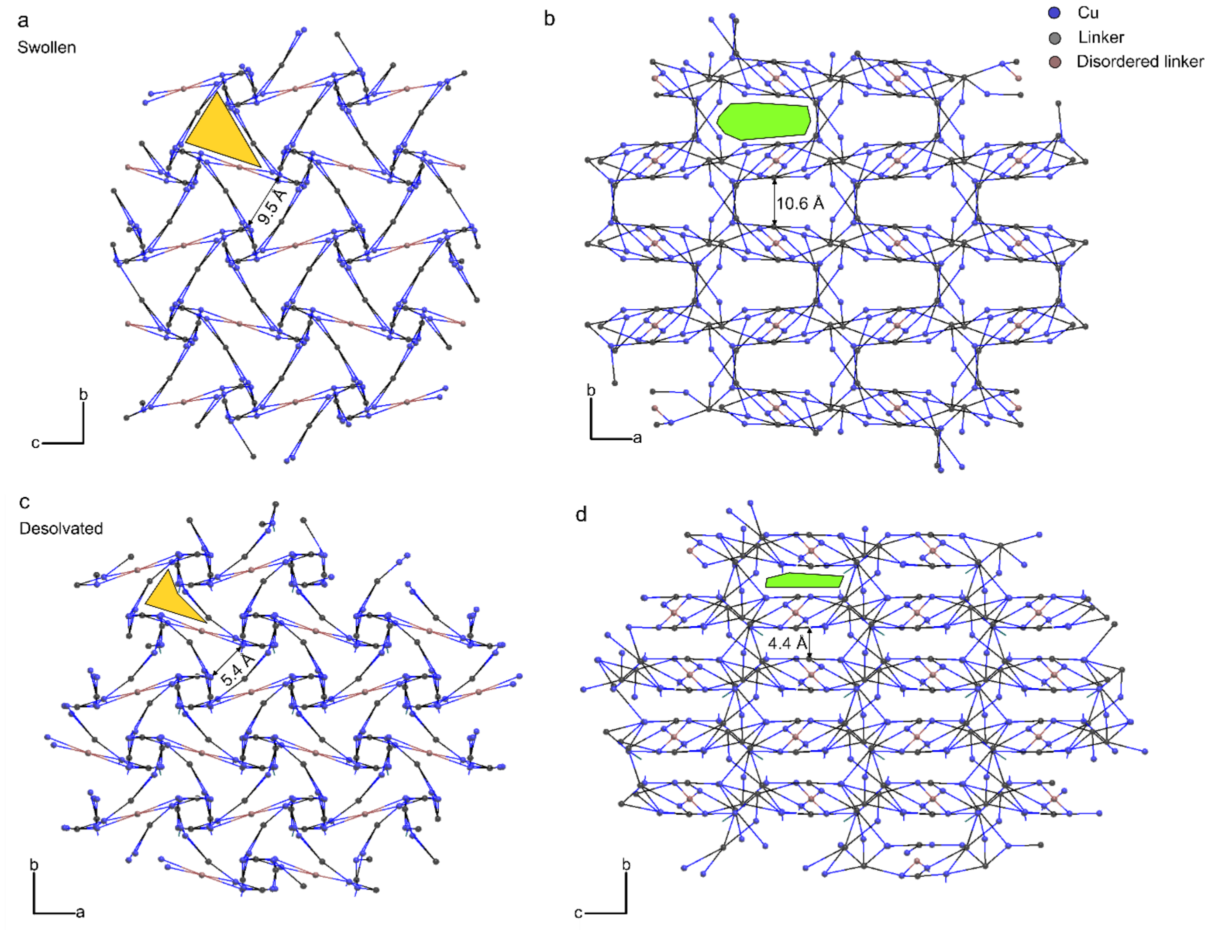

Figure S2: Simplified nodal representations of a) swollen SIMOF-5 down the x *axis, b) swollen SIMOF-5 down the* z *axis, c) desolvated SIMOF-5 down the* z *axis and d) desolvated SIMOF-5 down the* x *axis. Cu atoms are represented as blue spheres, DHTP as grey and disordered DHTP as brown. The triangular pore is highlighted in orange and the 'teardrop' pore in green.*

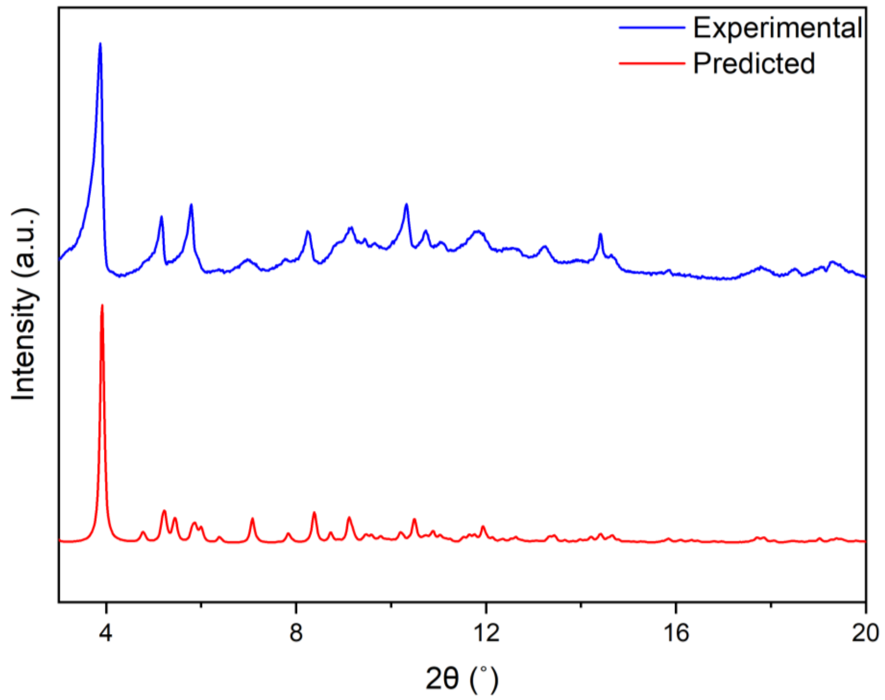

Figure S3: PXRD patterns of desolvated SIMOF-5 predicted from scXRD structure (red) and the experimental pattern (blue) (using MoKα radiation).

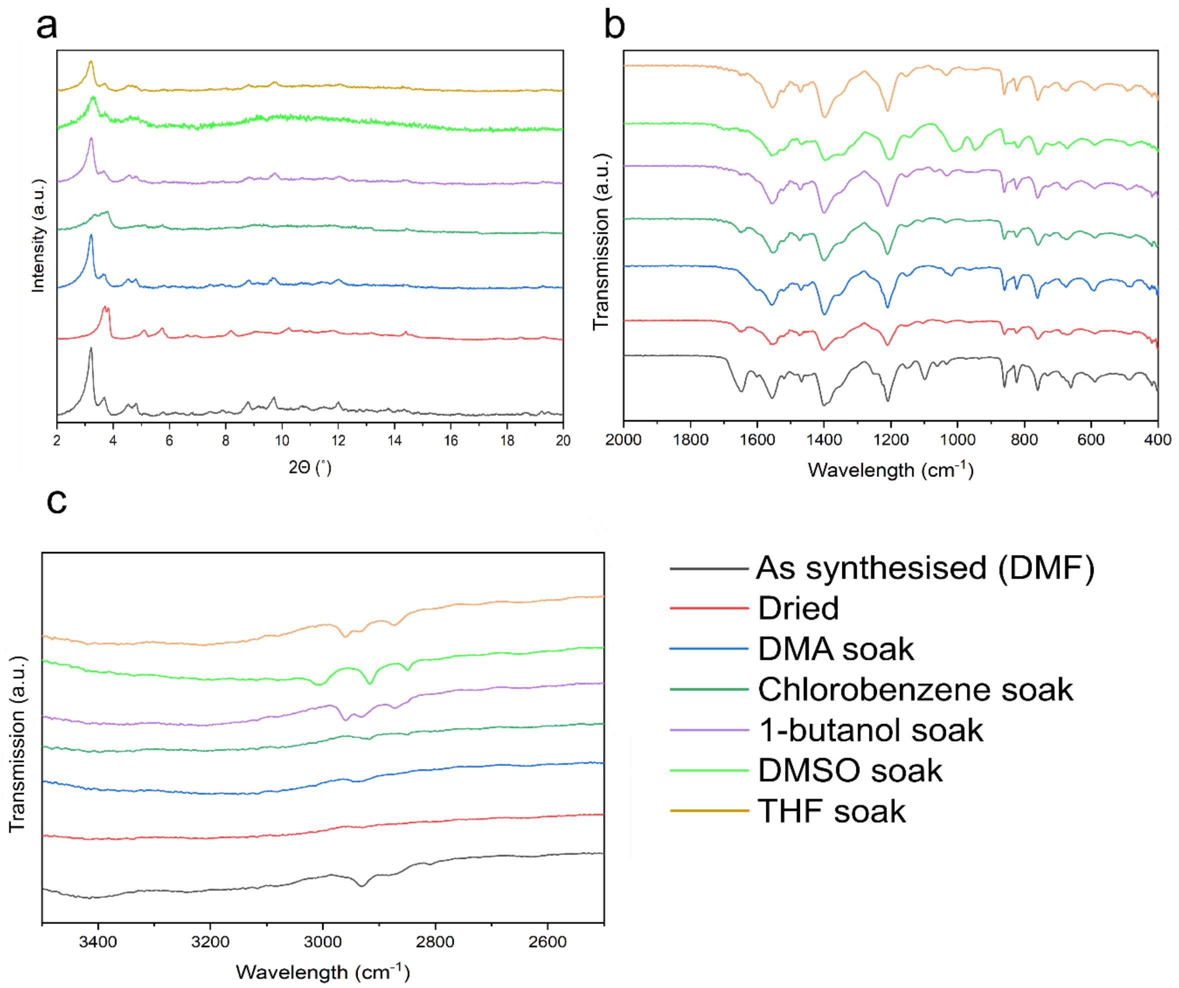

Figure S4: Showing a) PXRD patterns, b) and c) FTIR spectra of SIMOF-5 as made (black), solvent exchanged with EtOH and dried (red) and then soaked in: DMA (blue), chlorobenzene (green), 1-butanol (purple), DMSO (neon green) and THF (orange).

Table S1: Containing unit cell information on SIMOF-5 during different states of solvation and temperature. Obtained from PXRD data.

Sample	*a* length (Å)	*b* length (Å)	*c* length (Å)	*β* (˚)	Volume (Å^3)
scXRD solvated	17.071(3)	20.985(4)	16.560(3)	115.245(3)	5365(1)
Saturated in DMF	17.05(1)	21.96(2)	16.77(1)	114.65(5)	5709(5)
Dried at 60 ˚C for 10 min	17.09(1)	21.57(1)	16.69(1)	114.85(5)	5580(3)
At 100 K	17.05(1)	20.97(1)	16.68(1)	114.00(7)	5446(5)

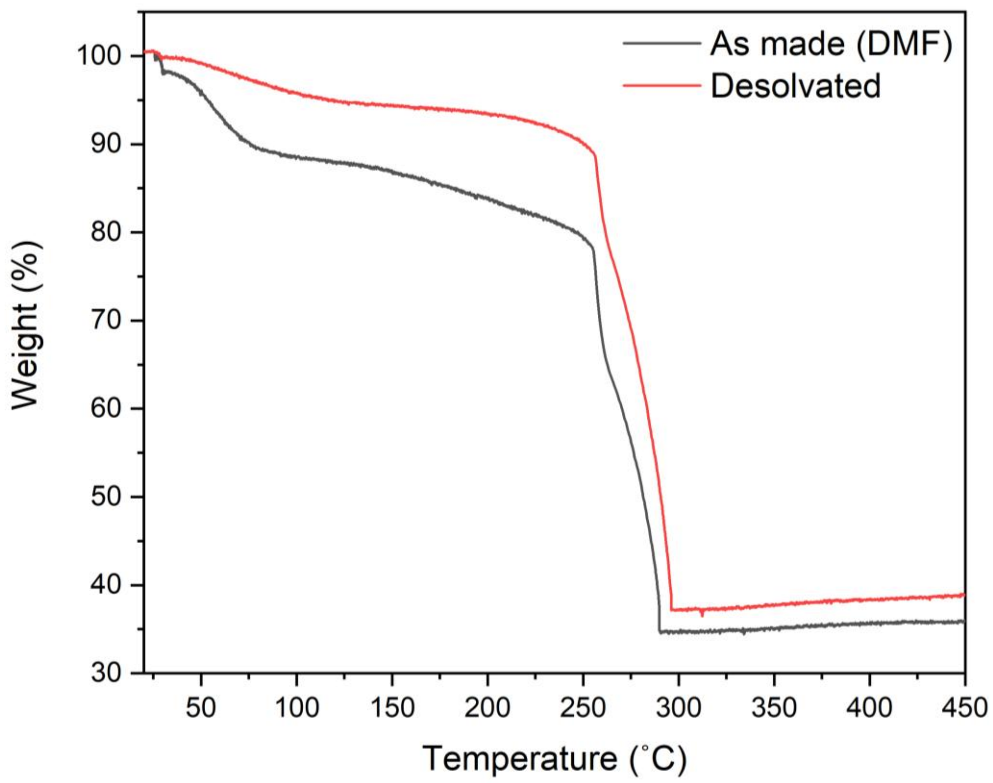

Figure S5: TGA data of SIMOF-5 in the as-made (black) and desolvated (red) forms. Data was obtained at a ramping rate of 5 °C/min under air flow.

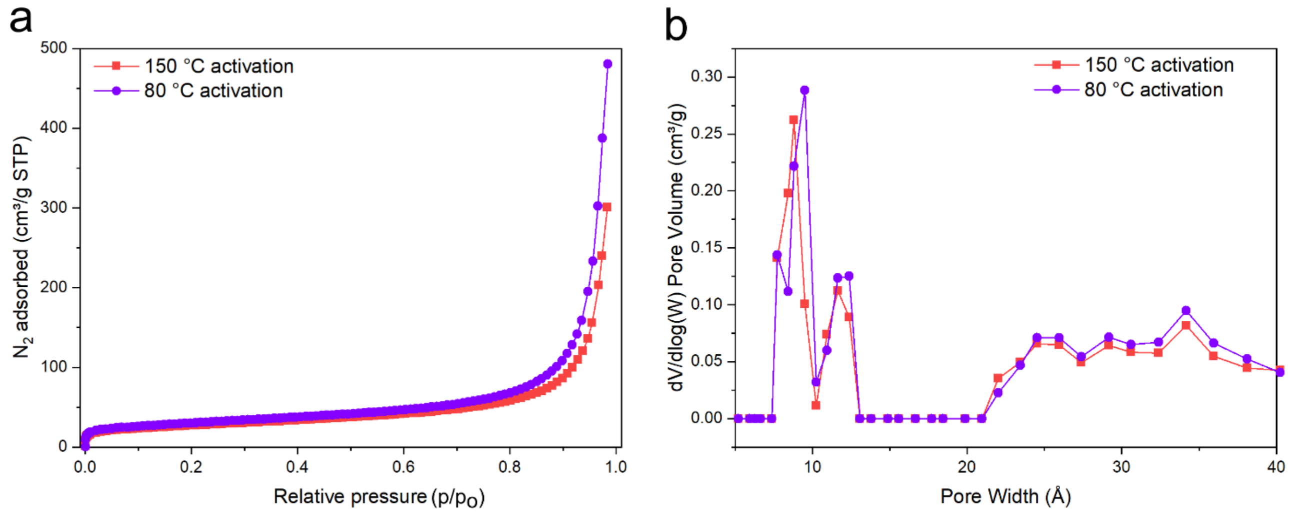

Figure S6: a) N_2 adsorption isotherm taken at 77 K for SIMOF-5 after activation under vacuum at 150 °C (red) and 80 °C (purple). Calculated BET surface areas of 91 and 102 m^2g^{-1} respectively. b) Pore size distributions as calculated using a NLDFT model. Low BET surface areas may be attributed to transition to the desolvated phase after activation.

Supplementary information on SIMOF-5 loaded with different molecules.

Table S2: Containing crystallographic unit cell information on SIMOF-5 loaded with different compounds.

Sample	*a* (Å)	*b* (Å)	*c* (Å)	α = γ (°)	β (°)	V (Å^3)
Benzoic acid@SIMOF-5	18.907(3)	19.2786(15)	40.468(2)	90	98.932(9)	14571.7
Ibuprofen@SIMOF-5	16.409(10)	23.396(8)	16.847(6)	90	118.78(6)	5668.73
Flutamide@SIMOF-5	16.715(6)	20.514(3)	16.066(4)	90	118.48(4)	4842.23

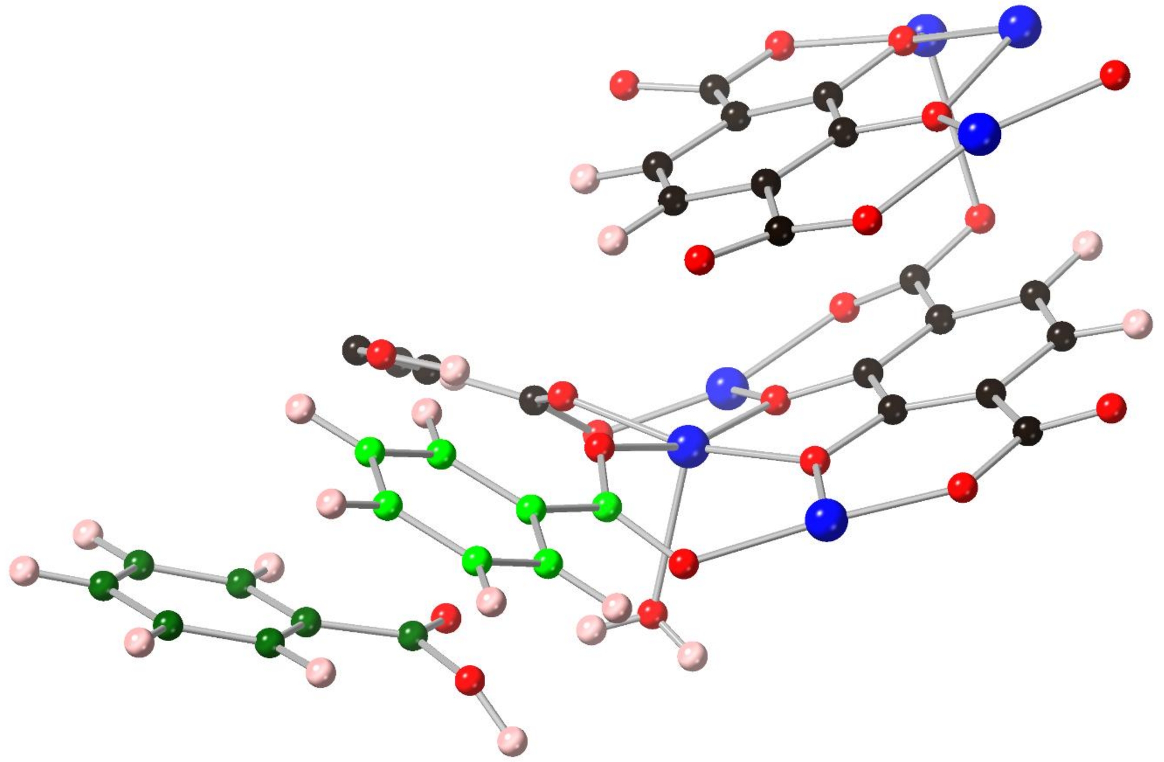

Figure S7: Ball and stick model of benzoic acid@SIMOF-5 showing the metal bound benzoate (light green) and the benzoic acid molecule in the pore (dark green). Key: Blue = copper, red = oxygen, black = carbon, pink = hydrogen. Note the carbon atoms of the benzoic acid molecules are changed.

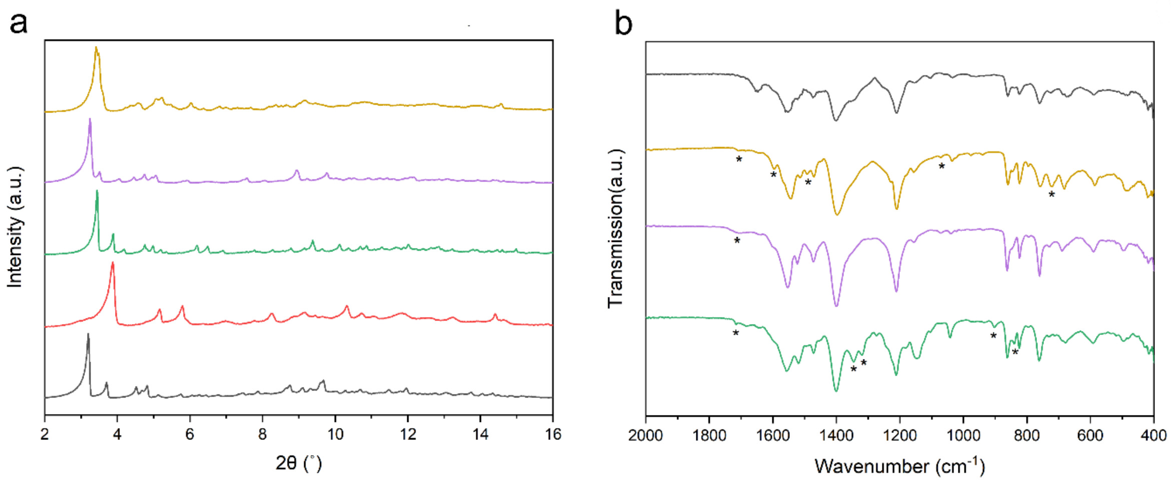

Figure S8: a) PXRD patterns and b) FTIR spectra of SIMOF-5 as made (black), desolvated (red), loaded with flutamide (green) ibuprofen (purple) and benzoic acid (yellow). Characteristic bands of the drug compounds are marked with asterisks in the FTIR spectra.

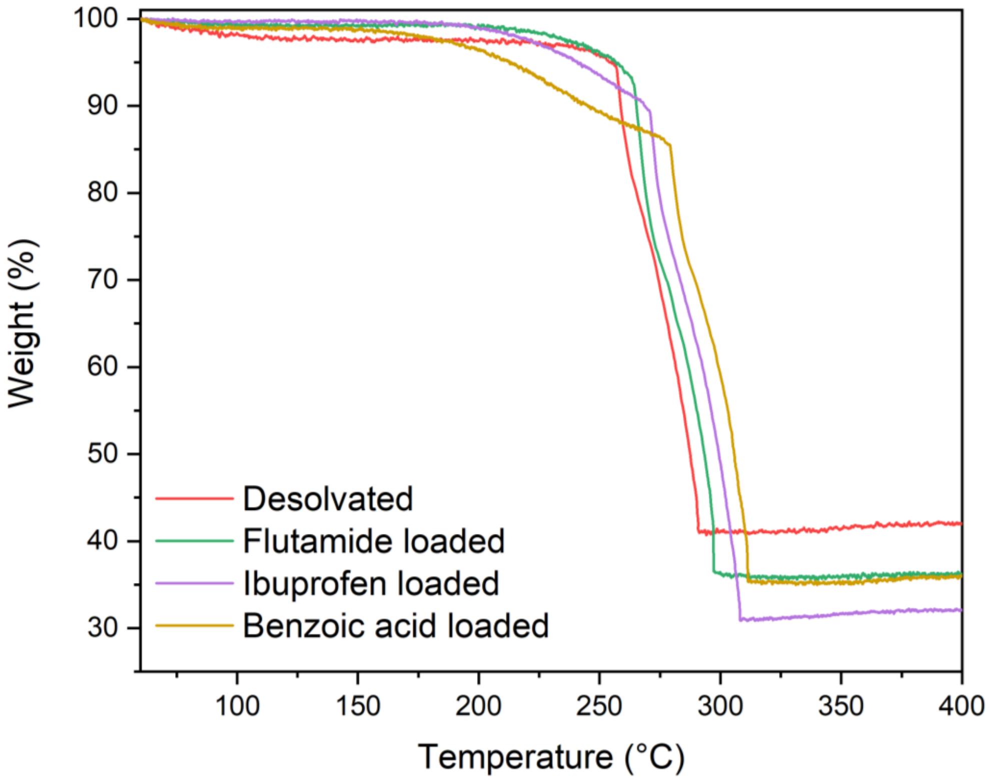

Figure S9: TGA data of SIMOF-5 desolvated (red) and loaded with flutamide (green), ibuprofen (purple) and benzoic acid (yellow). Data was obtained at a ramping rate of 5 °C min^{-1} under air flow.

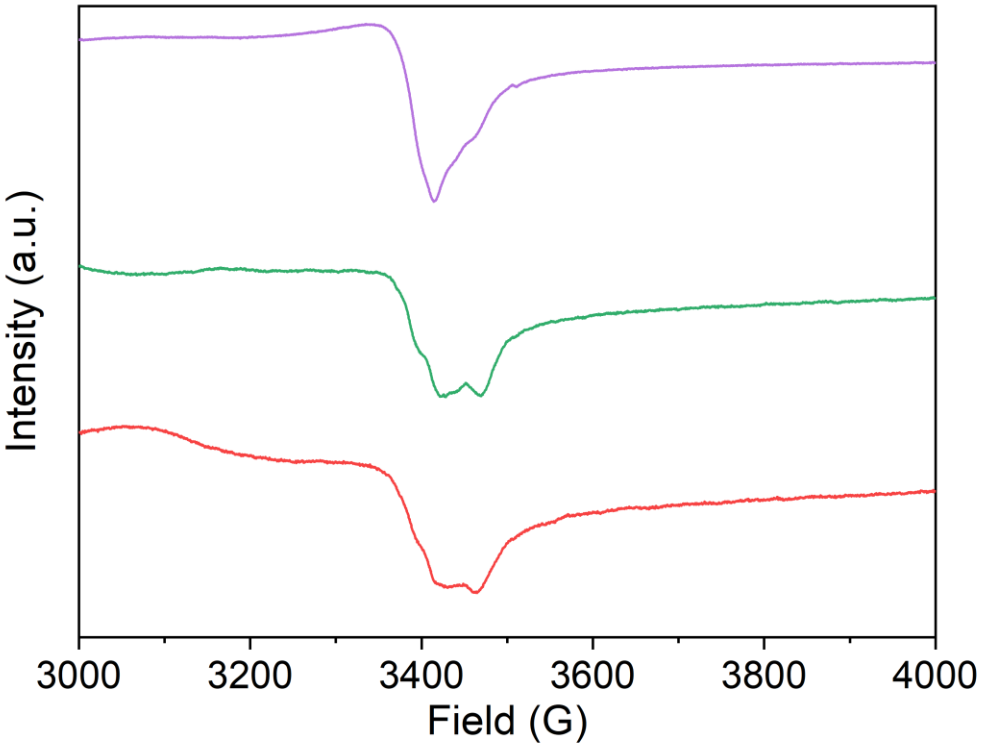

Figure S10: X-band EPR spectra of SIMOF-5 desolvated (red) and loaded with flutamide (green) and ibuprofen (purple) showing a significant change around the copper environment on binding Ibuprofen, but a smaller response to flutamide binding.

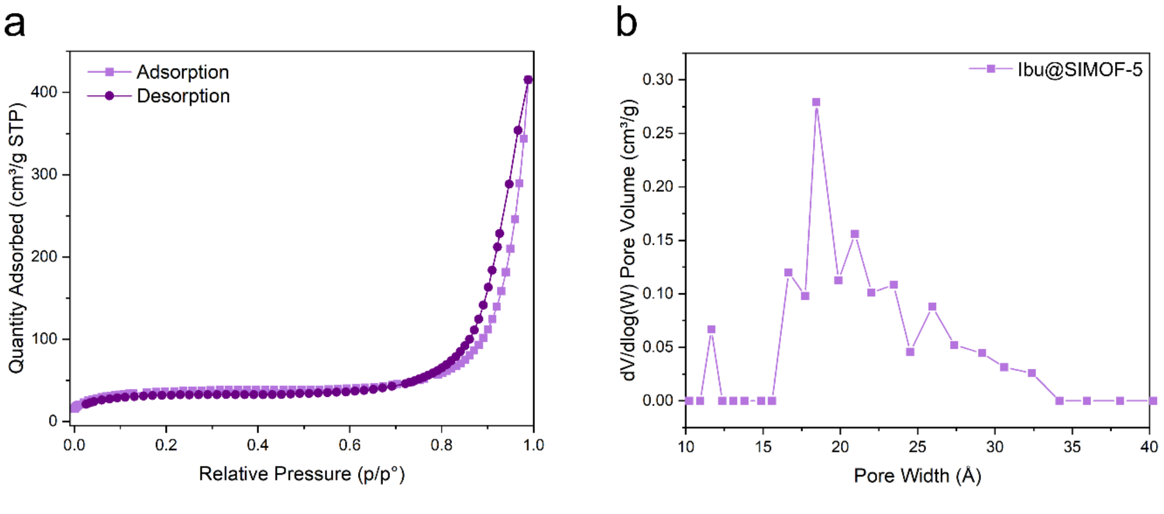

Figure S11: a) N_2 adsorption and desorption profile of Ibu@SIMOF-5 after activation at 80 °C under vacuum with a calculated BET surface area of 139 m^2g^{-1} b) Pore size distribution analysis of Ibu@SIMOF-5 calculated with a NLDFT model. The increase in surface area matches the crystal structure suggesting the pore is held open by ibuprofen binding.

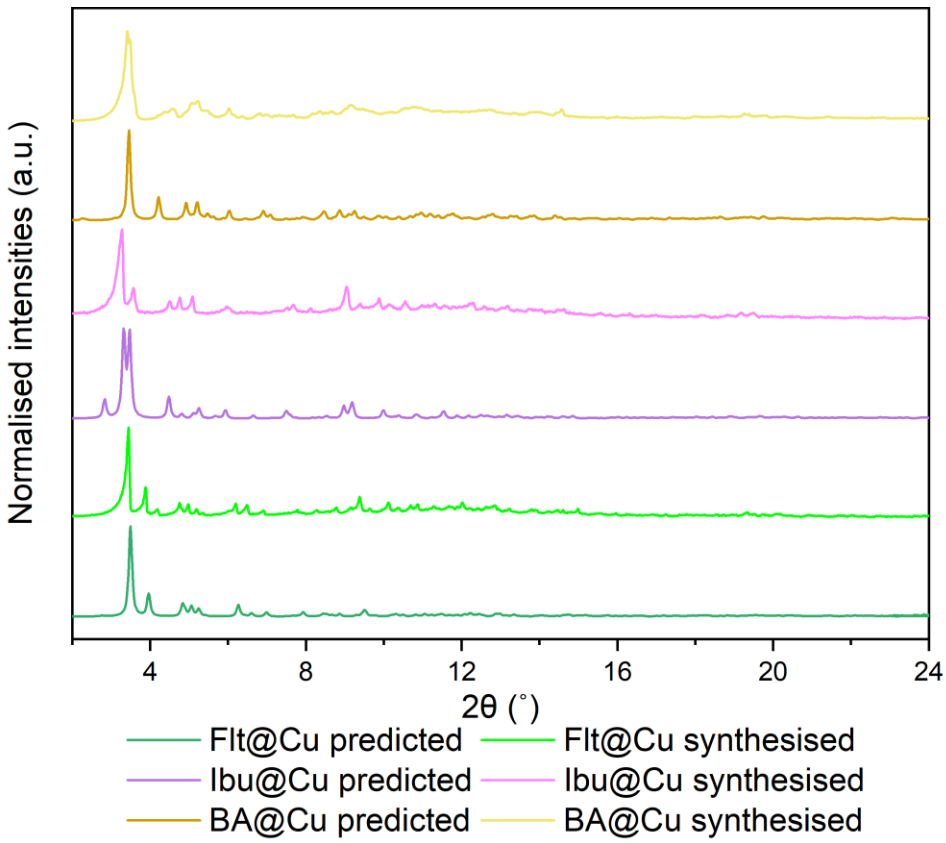

Figure S12: Showing powder patterns predicted from electron diffraction structures (dark) and as synthesised (light) for SIMOF-5 loaded with flutamide (green), ibuprofen (purple) and benzoic acid (yellow).

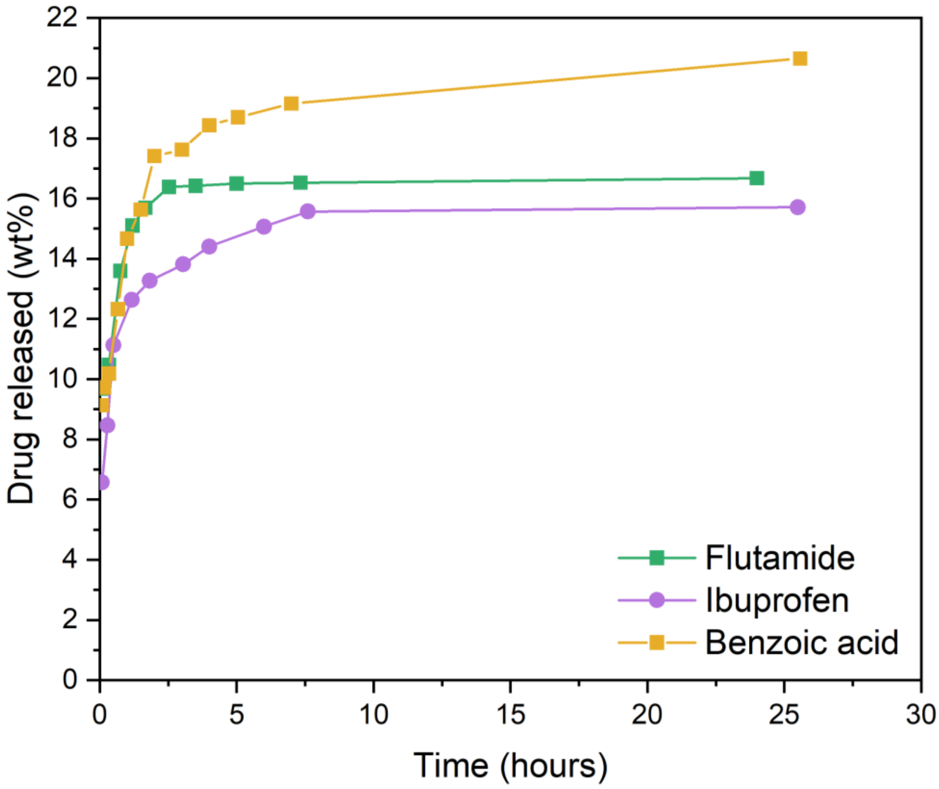

Figure S13: Drug release data from loaded SIMOF-5 in EtOH, as measured by UV/vis spectroscopy: flutamide (green), ibuprofen (purple) and benzoic acid (yellow), showing rapid burst release of flutamide and slower release of benzoic acid and ibuprofen.

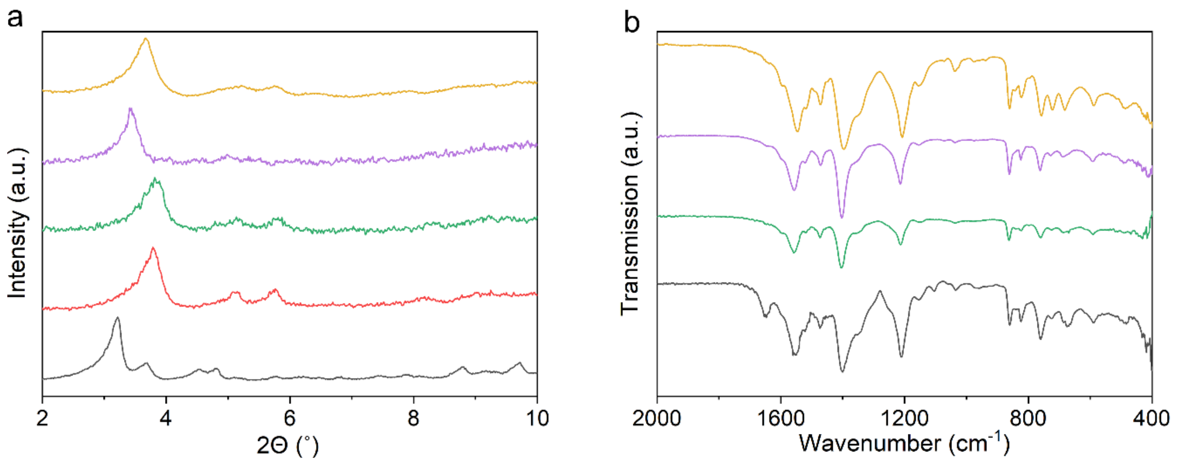

Figure S14: a) PXRD patterns of SIMOF-5 (black), dried (red) and after releasing, flutamide (green), ibuprofen (purple) and benzoic acid (yellow) and then drying at 60 °C. They show the structure has returned to the desolvated form. b) FTIR spectra of SIMOF-5 (black) and after releasing, flutamide (green), ibuprofen (purple) and benzoic acid (yellow) and then drying at 60 °C. There is no sign of bands associated with the loaded molecules.

Table S3: Table comparing the loading amounts of different molecules in SIMOF-5 as measured single-crystal diffraction, TGA and by release in EtOH as measured by UV/vis spectroscopy. The discrepancies between the values can be attributed to unmodeled molecules in the single-crystal structures, solvent in the TGA data and incomplete release into EtOH due to high binding energies.

Sample	Loading by sc diffraction (wt%)	Loading by TGA (wt%)	Loading by release in EtOH (wt%)
BA@SIMOF-5	22	19	20
Ibu@SIMOF-5	16	23	16
Flt@SIMOF-5	n/a	18	17

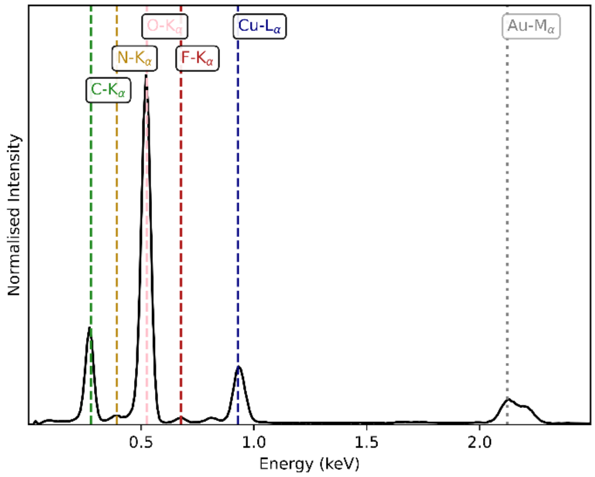

Figure S15: EDS spectrum obtained from the crystal used for structure determination of Flt@SIMOF-5.

Supplementary data on SIMOF-5@cotton

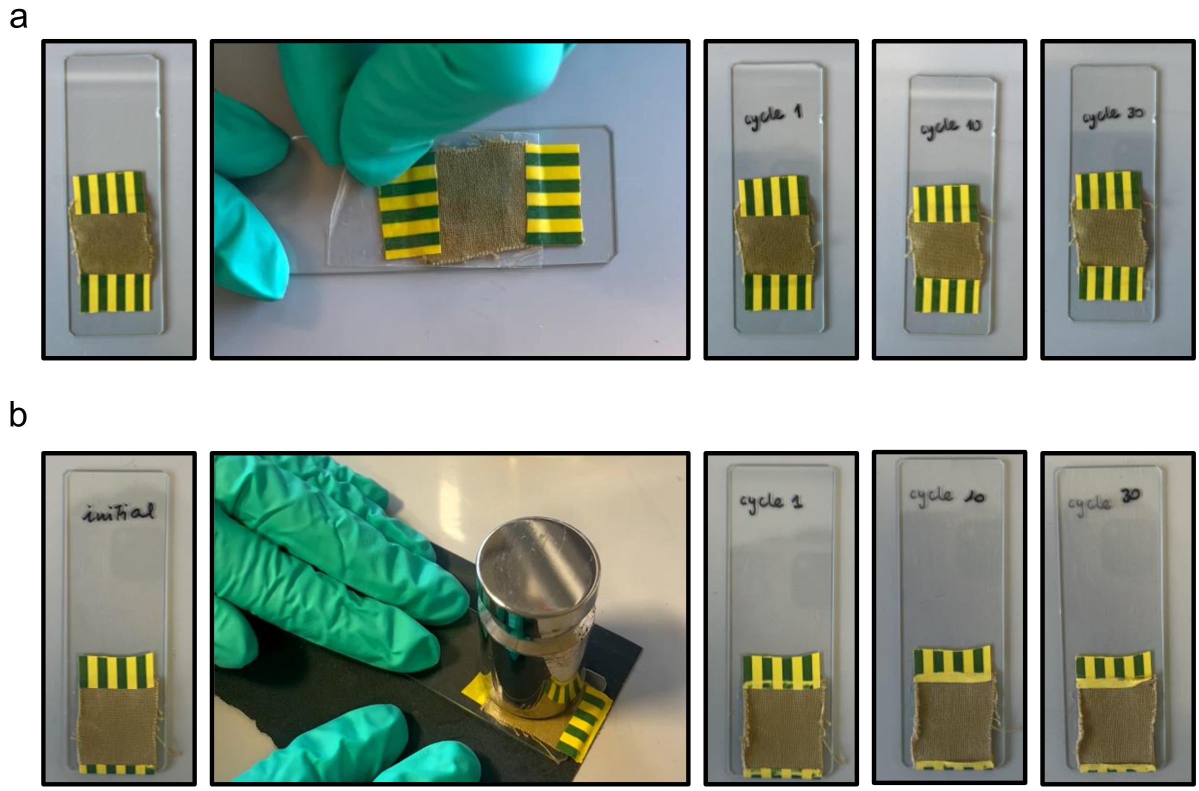

Figure S16: Images of a) adhesive peeling test and b) sandpaper test on SIMOF-5@cotton before and after 1, 10 and 30 cycles of abrasion.

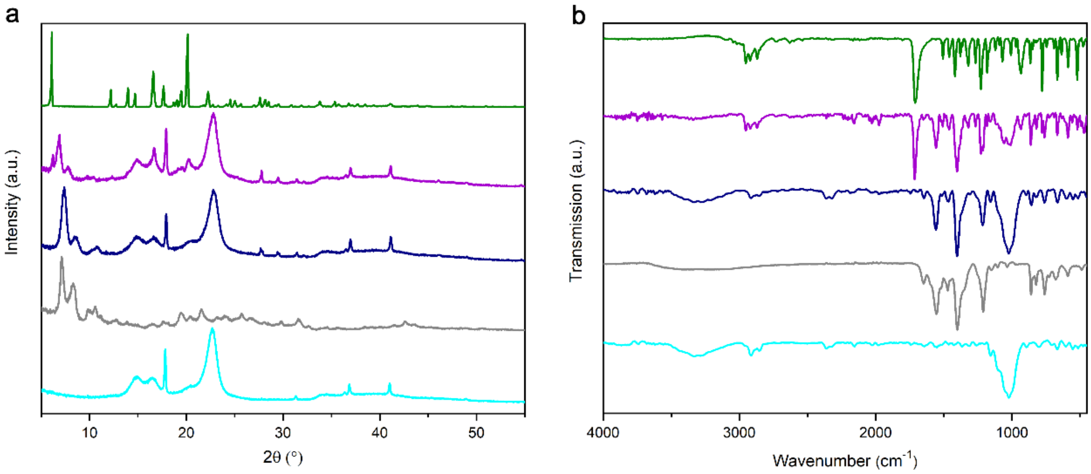

Figure S17: a) PXRD patterns, obtained with Cu Kα radiation, and b) FTIR spectra of cotton (light blue), SIMOF-5 as made (grey), SIMOF-5@cotton composite (dark blue), Ibu@SIMOF-5@cotton (purple) and ibuprofen (green).

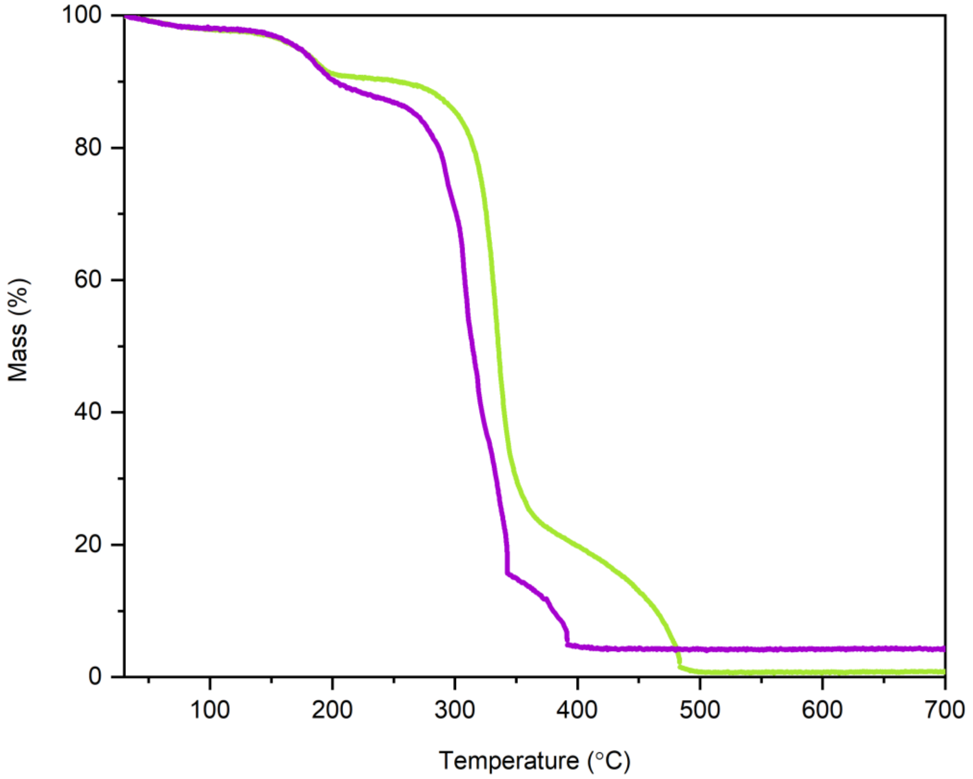

Figure S18: TGA data of Ibu@cotton (green) and Ibu@SIMOF-5@cotton (purple). Mass loss at 200 °C can be attributed to loss of ibuprofen.

Further refinement details

Table S4: Refinement details and CCDC deposition numbers for the five structures presented in this work.

Sample	Source	R_1	resolution (Å)	CCDC number
SIMOF-5 as made	Synchrotron X-rays	11.96	0.83	2404310
SIMOF-5 desolvated	Synchrotron X-rays	15.49	0.58	2404308
BA@SIMOF-5	Electrons	22.82	0.90	2415258
Ibu@SIMOF-5	Electrons	18.10	0.95	2415257
Flt@SIMOF-5	Electrons	23.38	1.00	2415259

Table S5: Crystal data and structure refinement for Ibu@SIMOF-5.

Identification code	Ibu@SIMOF-5 (CCDC 2415257)
Empirical formula	$C_{41}H_{27.77}Cu_6O_{23.39}$
Formula weight	1275.901
Temperature/K	175(5)
Crystal system	monoclinic
Space group	$P2_1/c$
a/Å	16.409(10)
b/Å	23.396(8)
c/Å	16.847(6)
α/°	90
β/°	118.78(6)
γ/°	90
Volume/Å^3	5669(5)
Z	4
ρ_{calc}g/cm^3	1.495
F(000)	789.8
Crystal size/mm^3	0.0007 × 0.00045 × ?
Radiation	electron (λ = 0.02510)
2θ range for data collection/°	0.12 to 1.52
Index ranges	$-16 \le h \le 16$, $-24 \le k \le 24$, $-16 \le l \le 16$
Reflections collected	15663
Independent reflections	5368 [R_{int} = 0.2381, R_{sigma} = 0.2753]
Data/restraints/parameters	5368/1476/658
Goodness-of-fit on F^2	1.233
Final R indexes [I>=2σ (I)]	R_1 = 0.1810, wR_2 = 0.4196
Final R indexes [all data]	R_1 = 0.3032, wR_2 = 0.4835
Min/max residuals	1.55/-1.13

Table S6: Crystal data and structure refinement for Flt@SIMOF-5.

Identification code	Flt@SIMOF-5 (CCDC 2415259)
Empirical formula	$C_{30}H_{23.33}Cu_6O_{27.67}$
Formula weight	1207.777
Temperature/K	175(5)
Crystal system	monoclinic

Space group	$P2_1/c$
a/Å	16.715(6)
b/Å	20.514(3)
c/Å	16.066(4)
α/°	90
β/°	118.48(4)
γ/°	90
Volume/Å^3	4842(3)
Z	4
ρ_{calc}g/cm^3	1.657
F(000)	704.1
Crystal size/mm^3	0.0024 × 0.0013 × ?
Radiation	electron (λ = 0.02510)
2θ range for data collection/°	0.12 to 1.44
Index ranges	$-16 \leq h \leq 16$, $-20 \leq k \leq 20$, $-16 \leq l \leq 16$
Reflections collected	12079
Independent reflections	4215 [R_{int} = 0.2930, R_{sigma} = 0.3251]
Data/restraints/parameters	4215/811/606
Goodness-of-fit on F^2	1.476
Final R indexes [I>=2σ (I)]	R_1 = 0.2338, wR_2 = 0.4855
Final R indexes [all data]	R_1 = 0.3063, wR_2 = 0.5219
Min/max residuals	1.50/-1.25

Table S7: Crystal data and structure refinement for BA@SIMOF-5.

Identification code	BA@SIMOF-5 (CCDC 2415258)
Empirical formula	$C_{123.93}H_{84.37}Cu_{18}O_{85.98}$
Formula weight	4092.958
Temperature/K	175(5)
Crystal system	monoclinic
Space group	$P2_1/n$
a/Å	18.907(3)
b/Å	19.2786(15)
c/Å	40.468(2)
α/°	90
β/°	98.932(9)
γ/°	90
Volume/Å^3	14572(3)
Z	4
ρ_{calc}g/cm^3	1.866
F(000)	2506.5
Crystal size/mm^3	0.0029 × 0.0023 × ?
Radiation	electron (λ = 0.02510)
2θ range for data collection/°	0.08 to 1.6
Index ranges	$-19 \leq h \leq 19$, $-21 \leq k \leq 21$, $-44 \leq l \leq 44$
Reflections collected	39418
Independent reflections	13988 [R_{int} = 0.2803, R_{sigma} = 0.3343]
Data/restraints/parameters	13988/2770/2144
Goodness-of-fit on F^2	1.311
Final R indexes [I>=2σ (I)]	R_1 = 0.2282, wR_2 = 0.4729
Final R indexes [all data]	R_1 = 0.3160, wR_2 = 0.5275
Min/max residuals	2.74/-1.62
